# Insights into the Relationship between Pentraxin-3 and Cancer

**DOI:** 10.3390/ijms232315302

**Published:** 2022-12-04

**Authors:** Maria Bogdan, Andreea-Daniela Meca, Adina Turcu-Stiolica, Carmen Nicoleta Oancea, Roxana Kostici, Marin Valeriu Surlin, Cristina Florescu

**Affiliations:** 1 Department of Pharmacology, Faculty of Pharmacy, University of Medicine and Pharmacy, 200349 Craiova, Romania; 2 Department of Pharmacoeconomics, Faculty of Pharmacy, University of Medicine and Pharmacy of Craiova, 200349 Craiova, Romania; 3 Department of Biochemistry, Faculty of Medicine, University of Medicine and Pharmacy of Craiova, 200349 Craiova, Romania; 4 Department of Toxicology, Faculty of Pharmacy, University of Medicine and Pharmacy of Craiova, 200349 Craiova, Romania; 5 Department of General Surgery, Faculty of Medicine, University of Medicine and Pharmacy of Craiova, 200349 Craiova, Romania; 6 Department of Cardiology, Faculty of Medicine, University of Medicine and Pharmacy of Craiova, 200349 Craiova, Romania

**Keywords:** cancer, pentraxin-3, inflammation, biomarkers

## Abstract

Although cancer can be cured if detected early and treated effectively, it is still a leading cause of death worldwide. Tumor development can be limited by an appropiate immune response, but it can be promoted by chronic extensive inflammation through metabolic dysregulation and angiogenesis. In the past decade, numerous efforts have been made in order to identify novel candidates with predictive values in cancer diagnostics. In line with this, researchers have investigated the involvement of pentraxin-3 (PTX-3) in cellular proliferation and immune escape in various types of cancers, although it has not been clearly elucidated. PTX-3 is a member of the long pentraxin subfamily which plays an important role in regulating inflammation, innate immunity response, angiogenesis, and tissue remodeling. Increased synthesis of inflammatory biomarkers and activation of different cellular mechanisms can induce PTX-3 expression in various types of cells (neutrophils, monocytes, lymphocytes, myeloid dendritic cells, fibroblasts, and epithelial cells). PTX-3 has both pro- and anti-tumor functions, thus dual functions in oncogenesis. This review elucidates the potential usefulness of PTX-3 as a serum biomarker in cancer. While future investigations are needed, PTX-3 is emerging as a promising tool for cancer’s diagnosis and prognosis, and also treatment monitoring.

## 1. Introduction

‘Cancer’ (neoplasm, malignant tumor) is a generic term naming more than 277 types of cancer disease which can affect any part of the body [1,2]. According to the World Health Organization, in 2020, cancer was responsible for nearly one in six deaths [2]. By 2040, it is estimated that there will be 28 million cancer cases and 16 million patients will not defeat the disease [3]. Cancer development implies both environmental and host factors and is a complicated process [4,5]. In women, the most common types of cancer are breast, colorectal, lung, cervical, and thyroid cancer, whereas in men, the most common types are lung, prostate, colorectal, stomach, and liver cancer [6]. Although in cases of solid tumors, the gold standard therapy is surgical resection, post-surgery cancer recurrence is common [7].

The connection between inflammation and cancer was first reported by Virchow in the 19th century and since then, the relationship has been extensively analyzed [8]. It has been shown that inflammation can support cancer cells proliferation and promote the tumor microenvironment through selectively blocking antitumor immunity, chronic inflammation being involved in almost all tumorigenesis stages [9,10,11]. Various inflammatory biomarkers have been discussed in cancer studies as they have been linked to advanced cancer stages, resistance to immunotherapy, and poor prognosis [12]: tumor necrosis factorα (TNF-α), interferon-γ (IFN-γ), and interleukin (IL)-1, IL-6, IL-8, IL-10, IL-11, and IL-17 [12,13,14,15,16,17,18,19].

Identified 30 years ago, pentraxin 3 (PTX-3) is a member of the pentraxin superfamily and an important inflammatory mediator [20,21]. This protein family has two subgroups: short (serum amyloid P component and C-reactive protein (CRP)) and long pentraxins (neuronal pentraxin receptor, neuronal pentraxin 1, neuronal pentraxin 2, PTX-3, and pentraxin 4) [22,23]. A 381-amino acid-long protein [24], PTX-3 is the prototype of long pentraxins and is expressed by non-immune and immune cells as a rapid response to inflammatory cytokines, microbes, and microbial components [25,26].It is essential for regulation of inflammation, innate immunity, angiogenesis, and formation, and remodeling of the extracellular matrix [27,28]. In healthy individuals, PTX3 is barely detectable in blood circulation, whereas its serum level strongly increases within 6–8 h of inflammation and infection [29,30].

**Figure 1:**
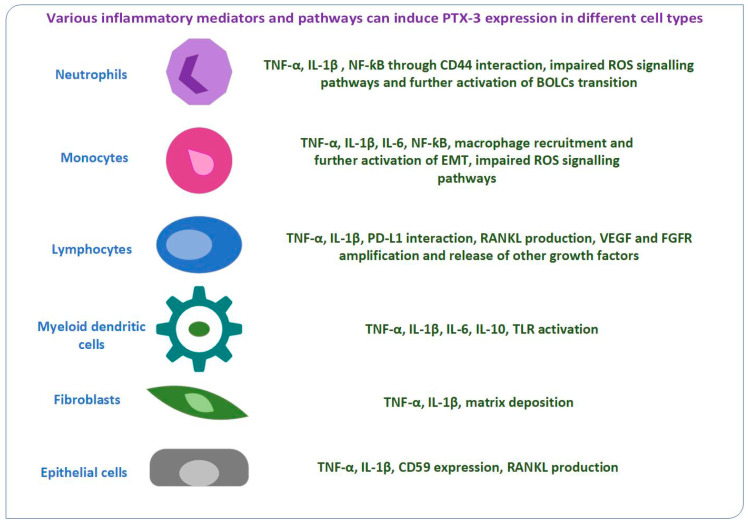
Increased synthesis of inflammatory mediators and activation of different cellular mechanisms can induce PTX-3 expression in various types of cells.

Figure 1 shows several immune regulators and pathways able to increase PTX-3 expression in malignancies.

PTX-3 is associated with many non-malignant conditions (e.g., asthma, hepatic cirrhosis, atherosclerosis, rheumatoid arthritis, systemic lupus erythematosus, sepsis, acute kidney injury, chronic kidney disease, stroke), but also it is overexpressed in various types of tumors (e.g., prostate/breast/lung/hepatic cancer, glioma) [31]. In this review, we present evidence for the correlation between PTX-3 and cancer, focusing on the underlying mechanisms of action.

## 2. Prostate Cancer

Hormonal signaling pathways have always been considered to play a principal role in developing prostate cancer, the second leading death cause that is diagnosed in men [32]. However, chronic inflammation has been associated in past years with a higher incidence of prostate cancer. The need to understand its molecular pathogenic mechanisms is increasing as more than 1.6 million new cases and 365.000 deaths are declared annually [33,34,35]. Prostate cancer progression has been linked with an immunosuppressive status induced by proliferating cells and, consequently, the inability of the human immune system to identify and to destroy neoplastic triggers [35].

Stallone et al. analyzed the potential of PTX-3 to discriminate tissue inflammation and benign prostatic hyperplasia from prostate cancer by evaluating both serum concentrations and tissular expression [36]. They further found both increased PTX-3 tissue expressions and serum concentrations in individuals who developed cancer in comparison with those diagnosed with prostatitis or benign prostatic hyperplasia. What is more interesting was the lack of differences in serum CRP and PSA levels in the patients included in their clinical study. In fact, serum PTX-3 curve proved better performance in identifying prostate cancer patients than the serum PSA curve [36].

Falagario and colleagues proved the out performance of PTX-3 accuracy in detecting prostate cancer in comparison with PSA. Their results underline the utility of PTX-3 as a discriminating factor of benign prostatic hyperplasia from cancer, at the cut-off value of 3.25 ng/mL (88.5% specificity and 89.3% sensitivity) and also as potential quantifying factor for prostatic cancer risk development [34].

Stallone et al. also assessed PTX-3 potential as a screening factor for disease progression. Even more, PTX-3 could be used as a target for the prevention of prostate cancer due to its predictive value (87.1% positive predictive value and 97.2% negative predictive value) [36].

Therefore, PTX-3 appears asa promising screening candidate as PSA, which is the only biomarker used until now in prostate cancer screening and is characterized by a high false positive rate and low accuracy [32,34]. These results were consistent with others from similar clinical studies where PTX-3 expression was increased in patients diagnosed with prostatic cancer in comparison with those diagnosed with prostatic benign hyperplasia [32,37]. Falagario et al. also concluded that PTX-3 has clinical utility as a prostate cancer predictor and could reduce the burden of multiple biopsies [34].

Unlike other pentraxin family members, such as CRP, PTX-3 is produced not by hepatocytes, but by different types of cells localized in inflammatory tissular areas [36], such as myeloid dendritic cells and peripheral blood leukocytes in response to TNF-α and IL-1β [35,38]. Prostate carcinogenesis is determined by chronic inflammation supported through cytokines and growth factors release, although CD8^+^Foxp3^+^ and CD4^+^CD25^+^ regulatory T cell concentrations increase in the peripheral blood of diagnosed individuals due to their immune suppressive activity [36]. Prostatic cancer cells also express specific regulatory proteins of the complement system [34]. On the other hand, PTX-3 sustains cellular migration and proliferation and dysregulates mitogenic signaling pathways; therefore, encouraging tumor escape from immune responses [34,36]. Moreover, PTX-3 ensures clearance of apoptotic cells after complement activating (through C1q interaction), suggesting a pro-neoplastic activity [32,34,36].

Interaction between the programmed death 1 (PD-1) receptor and the programmed death ligand 1 (PD-L1) on cytotoxic T lymphocytes represents one of the tumor-escape mechanisms activated in cancer cells. Increased expressions of PD-L1 have been linked not only with prostate cancer, but also with various malignancies such as colorectal, esophagus, lung, and renal cancer [35].

Scimeca et al. evaluated the relationship between PTX-3 and PD-L1 and found a significant inverse correlation in positive prostatic cancer cells [35]. Rasmussen et al. did not find a statisticallysignificant correlation between PTX-3 levels and risk of developing cancer in 197 patients with non-specific signs and symptoms, but noted a concluding association between CRP, ESR (erythrocyte sedimentation rate), suPAR (soluble urokinase plasminogen activator receptor), and newly diagnosed cancer [39]. These results underline the dual role of PTX-3 in tumor progression as its overexpression can be correlated with poor prognosis in individuals with gastric cancer, pancreatic cancer, breast cancer, glioma, and lung cancer, but its down-regulated expression can be found in esophageal cancer and melanoma [40,41,42,43,44,45].

The involvement of PTX-3 in complement cascade activation as a pathogenic pathway and its further insidious correlation with various types of cancers has been widely studied in the past years and results seem promising. PTX-3 is able to ensure escape of neoplastic cells from immunosurveillance mediated by complement system [32,36].

A single center cohort study conducted by Stallone and colleagues [32] proved intense interaction between C1q and PTX-3, increased expressions of two anaphylatoxins (C3a and C5a receptors) and tissular complement inhibitor CD59 in patients diagnosed with prostatic cancer while no interactions were noted in those diagnosed with prostatic benign hyperplasia. Up-regulation of CD59 is correlated with activation of terminal complement pathway, but PTX-3 restricts this pathogenic mechanism [32,34]. Both trans-membranary C3a and C5a receptors are essential in neoplasia development as they activate various signal transduction proteins (mammalian target of rapamycin, phosphatidylinositol 3-kinase, and Akt) and members of the mitogen-activated protein-kinase family (such as p38) [32,34]. Moreover, C3a and C5a activation lead to oxidative stress production that further restrains anti-tumor T cell and neutrophils responses [32]. Neoplastic cells tend to up-regulate complement receptors and bound complement inhibitors (such as CD59, CD55, and CD46) and Stallone et al. observed a clear correlation between increased values of CD59 and prostate cancer development [32]. CD59 expression is also induced by two of the most eloquent cytokines—IL-1β and TNF-α—in colon cancer cells. In other tumor contexts, C1q deposition through PTX-3 activation could lead to angiogenesis inhibition and reduced inflammatory responses [46,47]. Falagario et al. confirmed that adding the cost-effective PTX-3 as prostate cancer diagnostic biomarker to statistical models (along PSA) significantly increased the accuracy of disease detection and reduced the number of biopsies [34].

## 3. Breast Cancer

Decreasing the diagnostic time for breast neoplasm also involves avoiding the disease evolution while increasing rate of survival [39], therefore novel screening or diagnosis strategies are urgent. Worse prognostic features and diminished survival rate in triple-negative breast cancer (a disease already limited by high recurrence and inefficient therapeutic measures) have been associated with PTX-3 overexpression [48].

A study conducted by Choi and colleagues underlined the up-regulation of PTX-3 expression in 64 individuals diagnosed with breast cancer and also in distant bone metastases, which represent one of the most common metastases [44]. It is necessary to investigate why does this inflammation and humoral immune modulator lead to a higher rate of bone metastases rather than brain, liver, or lung. Higher levels of cytokines and chemokines (such as TNF-α, IL-1β, and IL-6), hypoxia and necrotic cellular death trigger recruitment of macrophages—precursors of osteoclasts [44]. PTX-3 acts as an osteolytic agent by promoting cellular migration, macrophage mobilization, RANKL production, and osteoclastogenesis [44,49].

Various microcalcifications patterns play an essential role in breast cancer development. Moreover, the statistical risk of diagnosing malignant disease is based on the number and properties of the microcalcifications, produced by breastosteoblast-like cells, further identified by mammography. Bonfiglio and colleagues obtained intensified PTX-3 signals in an environment with microcalcifications in comparison with those tissues without microcalcifications (collected from human breast biopsies), suggesting that PTX-3 could be used as a differentiation marker between malign and benign disease [49].

Another study conducted by Lee et al. correlated RANKL up-regulation (a precursor process for osteoblast synthesis) with higher levels of PTX-3 [50]. However, Bonfiglio et al. did not find a difference between RANKL levels in lesions with or without microcalcifications [49], suggesting that PTX-3 could present higher sensitivity and specificity rates as differentiation biomarker. On the other hand, inhibition of transcription factor NF-ƙB leads to blockage of PTX-3 up-regulation, indicating a possible pathogenic PTX-3 mechanism, as cytokines activate NF-ƙB in malignant cells [44,51,52]. Thomas et al. observed that NF-ƙB is necessary to increase PTX-3 expression and also that higher levels PTX-3 lead to promote NF-ƙB synthesis [53], underlining the bidirectional relationship between PTX-3 and NF-ƙB.

Moreover, Choi et al. analyzed whether or not bone metastatic cells in individuals diagnosed with breast cancer, over-expressed PTX-3 in comparison with non-bone metastatic cells. The researchers underlined the potential activity of PTX-3 as a prognostic biomarker in breast cancer [44]. On the other hand, in prostate cancer cell lines, higher levels of PTX-3 were expressed by non-bone metastatic cells, probably due to more frequent osteoblastic cellular activity rather than osteolytic activity (encountered in breast cancer cell lines) [44,54]. Once again, the dual role of PTX-3 is assembled depending on the type of process that is influenced [46].

PTX-3 is able to ensure migratory potential of malignant cells by stimulating their epithelial–mesenchymal transition (EMT) [44], a mechanism also seen in pancreatic cancer cells [55]. EMT is a peculiar plasticity of cancer cells and is described through self-renewal and self-differentiation further modulated by TGF-β1 (transforming growth factor) which further promotes cellular invasion, chemotherapy resistance, and metastasis [48,51]. Nevertheless, TGF-β1 has also been detected in pancreatic myofibroblast-like cells [48]. EMT involves cellular cytoskeleton rearrangements, polarity modulation, modifications in cellular adhesion, isolation, and apoptosis resistance and is often a reversible process [51].

Due to its capacity to bind to fibroblast growth factors (FGF such as FGF2 and GFG8b), the N-terminus part of PTX-3acts as an angiogenesis and cellular invasion inhibitor, as confirmed by studies performed on fibroblast growth factor-dependent murine melanoma and prostate cancer [46,48,56,57]. Therefore, PTX-3 could suppress tumor development in FGF-dependent cancers [48]. On the other hand, the C-terminus of PTX-3 (which contains a homologous domain similar to other pentraxin family members such as CRP) interacts with CD44 (a type I glycoprotein located on the surface of lymphocytes, neutrophils, macrophages), proving high affinity for this receptor and activates NF-ƙB signaling pathways in breast cancer cells leading to metastasis [48]. CD44 ensures signal transduction and remodeling of the tumor microenvironment, while NF-ƙB controls immune responses, cellular growth, and apoptosis, further generating EMT [51].

Scimeca et al. conducted a retrospective study where they analyzed and underlined the relationship between PTX-3 and CD44 through the NF-ƙB pathway by collecting 100 breast surgical biopsies [51]. Significantly, PTX-3, CD44, and NF-ƙb have multiple involvement pathways in bone metabolism and in osteoblast-like cells (BOLCs) transition in malignant breast disease [51], as other studies mentioned [49,50,53]. Therefore, the role of PTX-3 in pathogenic matrix remodeling in case of tissular injury can be expanded [53].

Nome et al. measured 17 serum inflammatory biomarkers in breast cancer patients who received chemotherapy at different time points in a phase II randomized clinical trial and obtained results that support PTX-3 as proangiogenic factor [58]. Their study reiterates the idea that PTX-3 could be used as a target in cancer types where FGF2 and angiogenesis are involved in pathogenesis (especially in hormone dependent tumors) [58], consistent with other studies [47,59].

Nevertheless, as various studies underline, due to PTX-3 cellular activity (autocrine/paracrine) similar with hormones, PTX-3 could serve as a malignant biomarker in diagnosis and prognosis evaluation [49,50,53]. Researchers remind that the importance of novel drug development in breast cancer and pipelines could be based on inhibitors of PTX-3/CD44 interaction or PTX-3 use in association with mesenchymal markers as the target [48,51]. However, notably, PTX-3 target therapies need to be correlated with genetic background [60,61,62].

## 4. Glioblastoma and Meningioma

Increased synthesis of inflammatory biomarkers and activation of chemokine receptors (mediated through IL-6, IL-8, CX3CL1), leukocyte and stromal cells recruitment, induction of reactive oxidative species, hypoxia, and necrosis have been also identified in glioblastomas—highly aggressive astrocytic tumors [45,63]. Glioma represents one of the most common and lethal tumors in middle-aged and elderly individuals with a poor prognosis due to rapid cellular infiltration and invasiveness [64,65]. Unfortunately, tumor recurrence appears despite tumor resection, intensive healthcare, and treatment [63,65]. Toll-like receptors (TLR) activation and transcription of NF-ƙB (emerged through prolyl-isomerase Pin1) also play an essential role in the inflammatory process within glioblastomas as cellular proliferation, migration, and apoptosis are further regulated [45,66]. Nevertheless, PTX-3 expression in gliomas is positively correlated with monocytes, fibroblasts, endothelial, and T cell levels [64]. Complement activation along with angiogenesis have also been noted in glioblastoma through PTX-3 modulation, similar to prostate and breast carcinomas [45]. Even more, through immunochemistry analysis, Locatelli and colleagues correlated malignancy with higher levels of PTX-3 glia-localized, produced by infiltrated macrophages in specimens collected from 63 patients diagnosed with primary central nervous system tumors [45].

However, PTX-3 was negatively correlated with IgG and B cell activation in the research conducted by Zhang et al., demonstrating inhibition of both immune and anti-tumor responses during cellular proliferation [64]. On the other hand, PTX-3 acts like a double-edge sword even in case of glioblastomas, as it promotes CD80 activation and Th1 differentiation [64]. Increased levels of regulatory T cells concomitant with reduced NK cells levels appear in patients receiving standard treatment (temozolomide and radiotherapy) after tumor resection [65,67,68].

Consequently, the roles of PTX-3 in cancer depend on the type of the tumor and microenvironment, as different signaling pathways are regulated through PTX-3 [64,69]. Various mechanisms are similar, as for example, Zhang et al. found that RELB (a critical regulon) could facilitate cellular proliferation and angiogenesis in both prostate cancer and gliomas and could also increase PD-L1 in prostate tumors [64,70].

PTX-3 values are differentially expressed in high and low-grade tumors (such as oligodendrogliomas and astrocytomas), as higher levels of TNF-α and IL-1β (sourced in necrotic cells) consequently increase PTX-3 levels, reflecting the ability of this humoral immune candidate to diagnose various types of malignancies [45,64]. TNF-α hyperactivates the NF-ƙB signaling pathway, promotes progressive evolution, insensitivity to treatment, and resistance to apoptosis [68], maintaining chaotic cellular division and invasiveness. Additionally, recurrent and secondary gliomas expressed higher PTX-3 levels in comparison with primary ones [64]. Interestingly, necrotic, and hypoxic areas as well as cells localized nearest to the vessel network produced factors further responsible for PTX-3 induction and angiogenesis [45]. Increased PTX-3 values lead also to a lower survival probability, as Zhang et al. concluded [64], consistent with other clinical studies [63,65]. Petterson et al. collected 18 tissues from patients diagnosed with glioblastoma and further used gene expression profiling in order to underline the importance of individual immunological profile in tumor recurrency [65].

Although Ke et al. found a statistical correlation between higher PTX-3 expression and shorter survival time in individuals with gliomas, they did not associate PTX-3 with poor prognosis in patients diagnosed with meningioma [63]. In their study, Ke and colleagues analyzed another transcription factor involved in essential cellular pathways (such as NF-ƙB, mTOR, and p53)—nuclear factor-like 2 (Nrf2), using paraffin-embedded tissues from 86 patients with gliomas and 111 patients with meningiomas [63]. Increased Nrf2 expression among with treatment resistance and poor prognosis was also noted in pancreatic and endometrial carcinomas [71,72]. In the clinical study conducted by Ke et al., PTX-3 and Nrf2 overexpressions determined worse outcomes in individuals diagnosed with glioblastoma in comparison with only one protein overexpression or none of the two biomarkers [63]. Therefore, not only that PTX-3 could be useful as a diagnostic tool, but also as a prognosis tool and it could support pathologists as a reliable high accurate predictor of various grades of gliomas and meningiomas, in correlation with genetic profile [63,65,73].

## 5. Gynecological Cancers

Similar to other inflammatory tumorigenic diseases, in response to TLR engagement and synthesis of TNF-α and IL-1β in gynecological cancers, PTX-3 is produced by monocytes, dendritic and endothelial cells, vascular smooth muscle cells, fibroblasts, adipocytes and macrophages [74]. Aboulouard et al. identified PTX-3 on a cohort of 41 human samples as a poor outcome gene marker in endometrial cancer, more specifically for grade I disease. Their results were obtained through in-depth proteomics analysis and are helpful in stratifying treatment decisions and guidance for professional healthcare [75].

Ishida et al. conducted a clinical study and investigated serum PTX3 involvement in endometriomas, uterine leiomyomas and mature cystic teratomas in 74 enrolled women. Their results proved that PTX-3 could be a useful diagnostic tool (with a cut-off index of >3.5 ng/mL) for mature cystic teratomas, one of the most common forms of ovarian cancer, as well as differentiating tools from endometriomas, which often present similar appearance on ultrasound scan [74]. Moreover, Ishida et al. proved positive PTX-3 immuno-staining in tumor specimens (such as epidermal tissues, sebaceous glands, and hair roots) [74], suggesting that PTX-3 increased expression may derive directly from the neoplasia and may further correlate with disease severity [76,77].

Berggrund et al. analyzed a possible diagnosis tool, through a proximity extension assay, consisting of 11-marker panel for HPV-positive (human papillomavirus) women and increased risk of developing invasive cervical carcinoma, the fourth most common cancer diagnosis in women worldwide and one of the first causes of death in women diagnosed with cancer [78]. After screening for cervical cancer through HPV identification, cytology is further recommended, although it has low sensitivity in discovering dysplasia that could progress to malignant tumors. Therefore, the need to appeal to multiple and more specific plasmatic biomarkers is urgent. In their clinical study, Berggrund and colleagues found a signature of 11 proteins (also including five proteins that have been previously associated separately with cervical cancer, such as PTX-3, IL-27, SIR2, proto-oncogene tyrosine kinase (SRC), and eukaryotic translation initiation factor 4E-binding protein 1 (4E-BP1)) with 0.96 sensitivity and 1.0 specificity, capable of distinguishing cases and controls [78]. Again, PTX-3 has been shown to play a role in microbe resistance [79,80]. Moreover, PTX-3 decreased plasmatic levels have been associated with suppression of metastatic potential of cervical cancer cells and lower possibility of HPV infection to evolve to dysplasia [79,80]. Although HPV tends to down-regulate NF-ƙB during persistent infections by altering the host immune responses, the signaling pathway is activated again in high grade cervical neoplasia [81,82,83], suggesting a possible correlation with PTX-3 and the other proteins from the signature panel [78].

Interestingly, Sun and colleagues found that PTX-3 genetic variants (rs1840680, rs2305619, rs3816527, and rs2120243) are not associated with tumorigenesis nor with 5 years survival of women diagnosed with cervical cancer, in a retrospective study that enrolled 548 women [84]. Yu et al. demonstrated involvement of micro RNAs (small non-coding RNA fragments) as regulators in the pathogenesis of cervical carcinoma after correlating inhibition of micro RNAs with PTX-3 gene in 132 enrolled women diagnosed with cervical carcinoma [83,85]. Increased miRNA levels have also been noted in other various types of cancers, such as hepatocellular, thyroid, medulloblastoma, and colorectal [86,87,88,89]. MicroRNAs have, on the other hand, potential anti-inflammatory activity through regulation of PTX-3 [90,91], but Yu et al. proved that over-expression of miR-224, a precursor of microRNAs, leads to cell progression and invasion of cervical cancer [83]. Increased values of miR-224 could also predict poor outcomes in patients diagnosed with prostate cancer [92]. Ying et al. underlined that PTX-3 expression is statistical significantly correlated with tumor grade and consequently, knockdown of PTX-3 directly suppresses metastasis in human cervical cancer cells [80].

Chang et al. obtained PTX-3 as the highest up-regulated gene in ovarian epithelial cancer tissues proving a definite clinical value of PTX-3 as both diagnosis and prognosis biomarker in ovarian epithelial cancer [93]. Yu and colleagues underlined that inhibition of miR-224 further targets PTX-3 gene and may prevent carcinoma progression in cervical tissue [83], therefore opening the road to researching potential therapeutic targets based on microRNAs and PTX-3.

## 6. Colorectal Cancer

Researchers have also investigated in the past years the usefulness of PTX-3 as prognostic predictor in colorectal cancer. Despite improvements in treatments and surgical techniques, the survival rate of individuals diagnosed with colorectal cancer is still poor [94]. Half of patients who are included in curative surgery for colorectal cancer will experience disease recurrence and death [95].

Zhang et al. included 400 patients in their clinical prospective study and evaluated PTX-3 plasma levels in correlation with cancer diagnostics. Values higher than 12 ng/mL on patients’ admission were associated with cancer relapse during the 60-month follow-up interval [96], although the pathogenic mechanisms are still incompletely understood. Nevertheless, PTX-3 levels decreased after tumor resection, but again increased in cases of recurrence [96], thus in cases of high-grade inflammation, hyperactive angiogenesis and inhibition of cellular apoptosis occurred, as other researchers have assessed [97]. Zhang et al. correlated complement activation with PTX-3, a similar mechanism with the one in prostate cancer mentioned above [96].

Epigenetic methylation of PTX-3 expression has been identified in colorectal cancer, revealing a possible onco-suppressor role of this acute phase protein [79,98]. Depending on tumor stage, the PTX-3 gene is methylated differently through two enhancers: enhancer-1 is silenced in initial colorectal cancer stages, while enhancer-2 is stimulated during carcinoma progression [79,98]. Therefore, as Giacomini et al. concluded, hypermethylation of different gene regions in PTX-3 is involved in both onset and progression of colorectal cancer [79].

Higher circulating levels of inflammatory cytokines predispose individuals to a greater risk of tumorigenesis [99,100]. Papila and colleagues compared serum values of NF-ƙB, PTX-3, IL-6, CRP, TNF-α, and sTRAIL (soluble TNF-related apoptosis-inducing ligand) and PCT (procalcitonin) in 40 individuals diagnosed with breast cancer, 40 with colon cancer, and 30 health controls [97]. In their clinical study, all markers were over-expressed in patients with cancer, except the serum sTRAIL values which were statistically significantly lower in those diagnosed with neoplasia in comparison with healthy ones. Moreover, NF-ƙB expression was positively correlated with PTX-3, PCT, CRP, and IL-6 in patients with breast cancer and colon cancer [97]. These results might suggest that combining NF-ƙB with PTX-3 or other inflammatory markers could lead to valuable prognostic scores in carcinomas [97], consistent with other clinical studies [101]. Interestingly, Papila et al. found that PTX-3 had the highest specificity and sensitivity in the cancer group, PTX-3 and PCT were significantly increased in colon carcinoma compared to breast neoplasia and that only PTX-3 was positively correlated with both IL-6 and NF-ƙB, suggesting that PTX-3 could be used as an independent prognostic tool for colorectal cancer [97]. These results are strengthened by the observational study conducted by Liu et al., as they found an optimal cut-off value for PTX-3 (12.6 ng/mL, 68% sensitivity and 71.7%, specificity) to identify colorectal cancer patients with a poorer 5-year overall survival rate [94].

DiCaro et al. found that TNF-α and IL-1β, although increased and strongly correlated in patients with colorectal cancer, were less associated with disease prognosis in comparison with PTX-3 [101]. Moreover, health professionals could assess patients with higher serum values of PTX-3 and could provide them with supplementary therapy or PTX-3 could also be considered a novel treatment target in case of colorectal cancer [94].

## 7. Gastric Cancer

Different mechanisms appear in gastric cancer, as PTX-3 promotes macrophage recruitment, cell invasiveness, and metastatic properties, while in esophageal squamous cell carcinomas and colorectal cancer, hypermethylation leads to PTX-3 gene silencing concomitantly with tumor growth [46,79,86,98]. Based on immunohistochemical analysis of human tumor specimens, increased PTX-3 hypermethylation was identified in the early stages of esophageal carcinogenesis rather than in advanced stages [79,86]. Various researchers have described the suppressing role of PTX-3 in cancer through complement regulation, as PTX-3 deficiency has been linked with increased susceptibility to tumorigenesis, dysregulated inflammation and signaling pathways, angiogenesis, enhanced macrophage differentiation, and disruption in modulation of host immune responses [46,79].

However, other researchers have mentioned that brain derived neurotrophic factor (BDNF) up-regulates PTX-3 expression and further stimulates osteoblastic interactions with gastric cancerous cells [43,79], making PTX-3 the main responsible biomarker for bone-derived metastases in patients diagnosed with gastric cancer. This is similar to PTX-3 osteolytic activity and enhancement of bone metastases in women with breast cancer. BDNF activates tropomyosin receptor kinase B (TrkB) and also RANKL production from osteoblasts, contributing to osteoclastogenesis [43,102,103]. After stimulating TrkB axis, BDNF enhances PTX-3 production, further responsible for aggressiveness and recurrence of gastric tumors [104,105,106], as Choi et al. concluded in an analyze of public cohort of 263 individuals from whom gastric cancer specimens were collected [43]. Elucidating pathogenic pathways and identifying novel biomarkers with the potential for usage in screening approaches in gastric cancer, could improve the survival rate for patients, as gastric cancer represents the fourth most common cancer type worldwide and the second leading cause of death (related to carcinoma) [107].

PTX-3 overexpression, infiltration of CD11b+ macrophages, TNF-α, and NF-ƙB activation were noted in human advanced gastric cancer tissues and contributed to gastric cancer-related inflammation [108]. Cytokines maintain a vicious cycle in gastric, pancreatic, and breast carcinoma [44,55] by up-regulating chemokine receptors and by promoting the ability of tumorigenic cells to migrate [108]. However, the precise mechanism of macrophage recruitment initially determined by PTX-3 increased levels is still not elucidated by researchers. The most recent clinical studies conducted by Cui et al. have underlined valuable results: PTX-3 low expression in gastric tissues is associated with macrophage polarization and stemness (represented by high capacity of invasiveness and EMT—epithelial–mesenchymal transition properties), through IL-4 and IL-10 secretion in the tumor microenvironment, while its increased expression inhibited migration of gastric cancer cell metastasis [109,110]. On the other hand, due to multiple associations between PTX-3 and gastric cancer progression through inflammatory pathways, PTX-3 could rather be considered a tumor promotor instead of tumor suppressor, especially in advanced stages or metastasis of gastric cancer [43].

Several pathways or molecules have been incriminated in gastric cancer development [107]; however, the connection with PTX-3 serum levels has not yet been realized in comparison with other types of cancer (such as prostate and breast tumors, colorectal cancer, and gliomas). For example, FGFRs signaling pathways interfere with cellular differentiation, proliferation, and invasion [107,111,112]. FGFR2 amplification has been linked with lymph node metastasis and a shorter survival term for patients diagnosed with gastric cancer [107,113,114]. VEGF also leads to neovascularization and activation of other growth factors; therefore, inhibition of VEGF/VEGFR activity could reduce gastric tumor proliferation by reducing hypoxia [115,116]. Yeni et al. found a statistical positive correlation between PTX-3 and VEGF, after evaluation of serum levels for both biomarkers in 45 individuals diagnosed with gastric adenocarcinoma and 30 healthy controls, but both biomarkers were significantly reduced in case of the patients group [116]. Therefore, the study conducted by Yeni and colleagues suggests a rather onco-suppressor activity of PTX-3 in gastric cancer [116], inconsistent with other studies [43]. Last but not least, numerous microRNAs have been involved in gastric cancer progression through the enhancement of oncogenes while reducing the expression of gene suppressors, and finally through EMT promotion into metastases [107,117,118,119]. It could be possible to correlate microRNAs with PTX-3 expression in gastric cancer similar to cervical cancer to determine the role of this acute phase protein in gastric adenocarcinomas. Further molecular and even genetic studies are urgent to explain the underlying mechanisms.

## 8. Pancreatic Cancer

Candidate biomarkers for early diagnosis in pancreatic cancers are crucial to be found as this type of cancer is frequently recognized too late [120,121]. PTX-3 is secreted and expressed in case of pancreatic cancer by pancreatic stellate cells (believed to be the precursors of carcinoma) [122,123,124,125]. Through a proteomic array analysis, Rosendahl et al. identified up-regulation of PTX-3 in pancreatic stellate cells from 55 evaluated patients [125]. During pancreatic tumorigenesis, the stellate cells become active after losing cytoplasmatic vitamin A-storing lipid droplets and after stimulating TGF-β and IL-6 synthesis [125,126]. These cells interfere with migration and deposition of extracellular matrix [125,127]. Preclinical studies have proven that targeting pancreatic stellate cells with ATRA (all-trans-retinoic acid) in combination with specific chemotherapy (gemcitabine-nab-paclitaxel) leads to tumor suppression, possibly by modulating PTX-3 expression [128,129].

Kamal et al. detected high levels of PTX-3 in early stages of pancreatic adenocarcinoma for 200 patients and also obtained good sensitivity and specificity values (0.63 and 0.85, respectively) for PTX-3 in proteomics analysis, suggesting therefore the quantification of serum PTX-3 as a reliable diagnostic biomarker [120]. Consistent with these results, Goulart et al. found a better predictive value (97%) for PTX-3 overexpression in diagnosing pancreatic cancer, with higher sensitivity and specificity (0.86 and 0.86) for values above 4.34 ng/mL [122]. However, in comparison with other types of cancer, PTX-3 serum levels were not different among patients with long or short survival rates [120]. Therefore, Goulart et al. proposed PTX-3 as a reliable stromal-derived diagnostic biomarker in patients with pancreatic ductal adenocarcinoma [122]. The sensitivity and specificity values for PTX-3 are more promising than those for the currently used serum biomarker (CA19-9) for pancreatic ductal adenocarcinoma diagnosis (sensitivity range 0.41–0.86 and specificity range 0.33–1 for CA19-9). Nevertheless, the study conducted by Goulart and colleagues demonstrated that PTX-3 serum levels are correlated with PTX-3 tissue levels in patients with pancreatic carcinoma and PTX-3 is presented as a differentiation biomarker between pancreatic cancer and other diseases (chronic pancreatitis and intraductal papillary mucinous neoplasm) [122].

On the other hand, Kondo et al. obtained higher values for PTX-3 in cases of individuals with advanced pancreatic cancer and shorter overall survival. Additionally, they underlined that the migratory activity of tumor cells is directly proportional with over-production of PTX-3 in pancreatic carcinoma cell lines [55]. In other words, PTX-3 correlates significantly with patient prognosis, similar to other types of cancer (such as prostate, breast, and ovarian) [79,130,131]. The regulation of matrix deposition and angiogenesis through PTX-3 in pancreatic cancer remains to be elucidated.

## 9. Lung Cancer

For lung cancer, the 5-year survival rates are only approximately 14% [132]; therefore, the need to identify potential and cost-effective diagnostic biomarkers is essential for patients. Nevertheless, PTX-3 can also be overexpressed in lung cancer cells and other pulmonary inflammatory diseases (such as bronchitis, asthma, and COPD) [39,133,134,135].

Zhang and colleagues achieved a large cohort study and measured PTX-3 serum levels in 1605 patients diagnosed with malignant and benign lung disease, further obtaining a diagnostic performance for this biomarker at an optimal cut-off value of 8.03 ng/mL (sensitivity and specificity of 72.8% and 77.3%, respectively) [134]. They also underlined that PTX-3 increased with tumor recurrence and decreased after tumor resection [134], consistent with other studies [132,135,136,137]. Infante et al. obtained over-expression of PTX-3 in both local and systemic levels in patients with lung carcinoma [136]. These results lead to the conclusion that PTX-3 could be used as a surveillance protein in order to assess treatment or surgical outcomes in individuals diagnosed with lung tumors [134].

Hu et al. found highly increased PTX-3 levels in bronchoalveolar lavage from patients with small cell lung tumor (with 81% sensitivity and 61.10% specificity for a cut-off value of 1933.0837 pg/mL). Moreover, their study demonstrated no statistical correlation between PTX-3 levels and age, gender, peripheral blood leukocyte, or neutrophil counts, concluding that PTX-3 expression in bronchoalveolar fluid could be a diagnosis biomarker for lung cancer [132]. However, several studies confirm that PTX-3 levels increased in more than 12 types of cancer, suggesting a non-specificity for this protein as a diagnostic marker in lung cancer [134,138].

As already mentioned, the inflammatory processes are the hallmark of tumor progression [135]. In the case of tobacco smoking, innate immune responses are activated with increased levels of TNF-α, neutrophils, and macrophages, with induction of DNA-mutations and, finally, with lung carcinogenesis [135]. Various proteins, such as PTX-3, ALCAM, and AXL, have been associated with metastatic lung tumors by EMT maintenance [135,139]. Liu et al. found a positive statistical correlation between PTX-3 up-regulation, male gender, smokers, lymph node metastasis, and reduced survival rate in individuals with small-cell lung carcinoma [140]. PTX-3 expression could also be targeted by anti-tumor treatment and could facilitate further development of drugs [140].

NF-ƙB activation and impaired reactive oxygen species (ROS) signaling pathways are also involved in lung cancer progression, similar to other types of cancer (such as colon, breast, pancreatic cancers, and leukemia) [39,107]. PTX-3 increases depending on ROS stimulation and leads to HIF-1α (hypoxia-inducible factor) and NF-ƙB accumulation. Both HIF-1α and NF-ƙB have the ability to bind to PTX-3 gene promoter and down-regulate RNA interference. Increased PTX-3 synthesis by lung cancer stem cells predisposes to invasive and metastatic properties. NF-ƙB stimulation is therefore linked with pro-inflammatory response, cellular migration and proliferation, apoptosis resistance and metastasis mechanisms and it could represent a target for anticancer chemotherapy by controlling PTX-3 expression through de-glycosylation when NF-ƙB pathway is blocked [141,142].

Nevertheless, targeting angiogenesis has also been a promising tool in fighting tumor progression in the past years. Although, angiogenesis mechanisms are still unclear, Gu and colleagues found, through bioinformatics methods and machine algorithms, a statistical positive correlation between over-expression of various angiogenic factors such as PTX-3 gene among other three genes (C1QTNF6, SLC2A1, and FSTL3) and non-small cell lung cancer [143]. In conclusion, PTX-3 serum and tissular levels represent valuable targets in lung cancer prognosis and diagnosis.

## 10. Hepatic Cancer

In hepatocellular tumors, PTX-3 has been correlated with a shorter survival time of patients [144,145]. Over-expression of PTX-3 increases macrophage chemotaxis, cellular proliferation, and EMT, therefore suggesting a pro-tumorigenic role in hepatocellular carcinoma [144,145,146].

Moreover, Deng et al. found a statistically significant correlation between higher PTX-3 levels and hepatocellular carcinoma and chronic hepatitis B virus infection in comparison with cirrhosis patients or healthy controls in their retrospective study [147]. PTX-3 can also be useful to differentiate chronic hepatitis B infections of early cancer, with a cut-off value of 9.231 ng/mL (79.4% sensitivity and 89.9% specificity) [147]. Identification of hepatocellular carcinoma at early stages is essential in order to increase survival rate. Nevertheless, PTX-3 can be a valuable tool to assess as a risk factor for tumor occurrence in patients diagnosed with chronic hepatitis C virus infection [148].

Carmo and colleagues found in a multivariate analysis that the PTX3 (rs2305619) A/A genotype was associated with hepatocellular cancer in patients infected with hepatitis C virus and that those individuals were twice predisposed to have carcinoma [148]. A/A genotype can activate multiple mediators that can sustain inflammatory response more than G/G or G/A genotypes, consequently leading more frequently to carcinoma diagnosis. PTX-3 is able to bind to bacteria and viruses and further activates the complement system, facilitating neutrophil and macrophage infiltration [148].

The complicated regulatory role of PTX-3 in hepatocellular cancer could be explained through TNF-α and IL-1β increased levels that further progress concomitant with the disease [145,147,149]. PTX-3 has been linked with metastatic risk as it induces EMT in hepatocytes, promotes fibrocytes differentiation and supports cellular invasion [147,148]. On the opposite, Feder et al. found no association between PTX-3 circulating levels and disease severity in individuals diagnosed with hepatocellular carcinoma, but they included a limited cohort in their study as they analyzed the serum of 31 patients [150]. This protein should be compared in future clinical studies with other non-invasive biomarkers in large cohorts to better establish its diagnostic value in hepatocellular carcinoma. It has been considered a non-invasive biomarker in cancer as it can be rapidly detected from serum without requiring tumor resection [150,151].

## 11. Renal Cancer

PTX-3 can also be useful as a non-invasive serum biomarker for diagnosing and anticipating prognosis of renal cancer. A 10-year prospective cohort study conducted by Netti et al. obtained elevated PTX-3 levels in renal cancer cell lines and tissues correlated with metastasis and statistically significant lower survival rates, concomitant with no PTX-3 activity in patients with normal renal proximal tubular cells and normal renal tissues [151].

The complement system, as part of the innate immune pathways, stimulates phagocytic cells and antibodies to clear damaged cells from an organism by altering the cell membrane of the pathogen and promotes further inflammatory responses by releasing pro-angiogenic anaphylatoxins (C3a and C5a). Although experimental data regarding complement activation in cancer are limited, the complement system (through cell proliferation and regeneration) has been linked in the past years by researchers with cancer progression, including renal cancer [151]. PTX-3 activates the C1q pathway and increases C3a and C5a activity while inhibiting cellular lysis through CD59 (also called protectin) up-regulation. Therefore, inhibition of complement cascade plays an essential role in pro-tumorigenic cells escape from immunosurveillance. Both receptors, C3aR and C5aR, have been specifically identified in metastatic renal cell tumors. Nevertheless, Netti et al. statistically associated PTX-3 over-expression with a lower survival rate [151].

On the other hand, Urquidi et al. concluded that monitoring PTX-3 levels could only be useful for evaluation of individuals diagnosed with bladder cancer, and not for diagnosis [152]. The FGF/FGFR system can be impaired and several fibroblast-growth factors (FGF1, FGF2, FGF5, and FGF8) are increased in human bladder carcinoma [153,154]. Through FGR pathway, EMT is stimulated and cellular invasion is maintained [47,153,155]. Interestingly, PTX-3 can inhibit FGF-dependent EMT and can hamper tumor progression [153]. PTX-3 exerts an onco-suppressive role in urothelial cancer, as in vitro and in vivo studies confirm [47,156,157]. These results underline the dual role of this acute phase protein [153].

## 12. Hematologic Malignancies

PTX-3 is produced by endothelial and myeloid cells and increases even more in chronic lymphocytic leukemia. As PTX-3 gene promoter binds to STAT3 which is also activated in leukemia, down-regulation of PTX-3 could stimulate apoptosis of invasive cells. In other words, PTX-3 inhibition may support patients with leukemia [158].

A study conducted by Brunel and colleagues demonstrated a correlation between PTX-3 polymorphism and the risk of developing mold infections in individuals diagnosed with acute leukemia [159]. An independent predictor for invasive infections was represented by homozygosity for the minor allele of PTX3 rs2305619 and/or rs3816527 in patients with neutropenia [159]. Neutrophils are responsible for both production and storage of PTX-3 and neutropenia could lead consequently to impaired immune mechanisms as PTX-3 ability to opsonize microbial surfaces would be hampered [160,161].

Herrero-Sánchez et al. also showed a strong association between PTX-3 gene polymorphism and risk of fungal infections in 198 patients diagnosed with hematological malignancies [161]. These researchers affirmed the importance of PTX-3 expression in establishing proper antifungal prophylaxis in immunosuppressed individuals [159,161,162]. Therefore, PTX-3 appears as a reliable marker in monitoring patients with leukemia [159].

Mechanisms of action, involvement of PTX-3 in various types of neoplasm and reported cut-off values are summarized in Table 1.

## 13. Conclusions

In 2022, 609,360 cancer deaths are predicted in the United States of America [163] and 1,269,200 cancer deaths in the European Union [164]; therefore, cancer research is important for effective prevention; earlier diagnosis; delivering safer, better, and potentially more affordable therapies [165]. Although inflammatory host response represents an important weapon in various physiological situations, when it becomes chronic, it may also contribute to cancer pathogenesis. The same biomarkers can be involved in both tumor progression and defence, showing tremendous complexity. PTX-3, yet poorly understood, seems to be a pioneer in regulating cancer in both ways: it could settle an initial proper immune and inflammatory response and subsequently inhibit tumor progression, or it could increase metastatic risk. PTX-3 is emerging as a promising non-invasive tool for cancer diagnosis, evaluating prognosis, monitoring chemotherapy, and novel management strategies. Further in-depth studies and larger cohorts of patients are needed to reinforce this status of PTX-3.

## Figures and Tables

**Table 1:** PTX-3—mechanisms of action and involvement in various types of cancer.

Type of Cancer	Mechanismof Action and Involvement of PTX-3 in Cancer	Cut-Off Value
Prostate cancer	- Pro-neoplasic activity by ensuring clearance of apoptotic cells (through PD-1 and PDL-1 interaction) after complement activation [32,34,35,36]; - Interacts with C1-q and increases C3a and C5a expressions [32,34]; - PTX-3 restricts CD59 up-regulation [32,34].	3.25 ng/mL (88.5% specificity and 89.3% sensitivity), proving utility as discriminating factor of benign prostatic hyperplasia from prostate cancer [34]
Breast cancer	- Acts as an osteolytic agent by promoting cellular migration, macrophage mobilization, RANKL production and osteoclastogenesis, increasing metastasis risk [49,50]; - Stimulates EMT [44]; - Bidirectional relationship between PTX-3 and NF-ƙB (through CD44 interaction) [48,53]; - Binds to various FGFs and acts as a potential angiogenesis inhibitor–potential suppressor of tumor development [48,56,57].	-
Glioblastoma and meningioma	- Complement activation and angiogenesis [64]; - Inhibition of host anti-tumor and immune response by decreasing IgG and B cell synthesis [64].	
Gynecological cancer	- Stimulates metastatic potential of cervical cancer cells and increases possibility of HPV infection to evolve to dysplasia [79,80]; - Targeting PTX-3 gene can lead to inhibition of micro RNAs in women diagnosed with cervical carcinoma [83,85]; - Knockdown of PTX-3 directly supresses metastasis in human cervical cancer cells [80].	PTX-3 could be useful as both diagnostic and differentiating tool (>3.5 ng/mL) for mature cystic teratomas from endometriomas [74]
Colorectal cancer	- Hypermethylation of different gene regions in PTX-3 is involved in both onset and progression of colorectal cancer [79]; - Positive correlation between PTX-3 and NF-ƙB [97]; - PTX-3 levels decreased after tumor resection, but again increased in case of recurrence (characterised by high-grade inflammation, hyperactive angiogenesis and inhibition of cellular apoptosis) [96,97].	At 12.6 ng/mL (68% sensitivity and 71.7% specificity) PTX-3 can be used to identify colorectal cancer in patients with poorer 5 years overall survival rate [94]
Gastric cancer	- Promotes macrophage recruitment, cell invasiveness and metastasis [46,79,86,98]; - BDNF up-regulates PTX-3 expression and further stimulates osteoblastic interactions with gastric cancerous cells, contributing to osteoclastogenesis [43,79]; - Positive correlation between PTX-3 and VEGF, suggesting an onco-suppressor activity of PTX-3 in gastric cancer [116]; - Possible correlation between microRNAs and PTX-3 [107,117,118,119].	-
Pancreatic cancer	- PTX-3 serum levels are correlated with tissular levels [122]; - PTX-3 could be a reliable differentiation biomarker between pancreatic cancer and other diseases (chronic pancreatitis and intraductal papillary mucinous neoplasm) [122]; - Migratory activity of tumor cells is directly proportional with over-production of PTX-3 in pancreatic carcinoma cell lines [55,79].	4.34 ng/mL (86% sensitivity and 86% specificity) for neoplasia diagnose [122]
Lung cancer	- PTX-3 sustains EMT [135,139]; - PTX-3 expression increases depending on ROS stimulation and leads to HIF-1α and NF-ƙB accumulation; both further bind to PTX-3 gene promoter and down-regulate RNA interference [141,142]; - Involvement in angiogenesis, not yet elucidated [143].	8.03 ng/mL (72.8% sensitivity and 77.3% specificity) [134]
Hepatic cancer	- PTX-3 increases macrophage chemotaxis, cellular proliferation and EMT (pro-tumorigenic role) [144,145]; - PTX3 (rs2305619) A/A genotype was associated with hepatocellular cancer in patients infected with hepatitis C virus and increased the risk to have carcinoma twice [148]; - Induces increased levels of TNF-α and IL-1β [145,147,149]; - PTX-3 has been linked with metastatic risk as it activates EMT in hepatocytes, promotes fibrocytes differentiation and supports cellular invasion [147,148].	9.231 ng/mL (79.4% sensitivity and 89.9% specificity)—useful to differentiate between chronic hepatitis B infections and early cancer [147]
Renal cancer	- Elevated PTX-3 levels in renal cancer cell lines and tissues correlated with metastasis [151]; - PTX-3 activates C1q pathway and increases C3a and C5a activity while inhibiting cellular lysis through CD59 up-regulation [151]; - PTX-3 can act as onco-suppressive protein in urothelial cancer by inhibiting FGF-dependent EMT [153,156,157].	-
Hematologic malignancies	- PTX-3 gene promoter binds to STAT3 and down-regulation of PTX-3 could stimulate apoptosis of invasive cells [158]; - Positive correlation between PTX-3 polymorphism and the risk of developing mold or fungal infections in individuals diagnosed hematologic malignancies [159,161].	-

## Data Availability

Not applicable.

## Abbreviations

4E-BP1	Eukaryotic translation initiation factor 4E-binding protein 1
Akt	Serine/threonine protein kinase (protein-kinase B)
ALCAM	Activated leukocyte cell adhesion molecule
ATRA	All-trans-retinoic acid
AXL	Member of receptor tyrosine kinases family
BDNF	Brain-derived neurotrophic factor
BOLCs	Osteoblast-like cells
CD44	Type I glycoprotein
CD59	Protectin
CRP	C-reactive protein
EMT	Epithelial–mesenchymal transition
ESR	Erythrocyte sedimentation rate
FGF	Fibroblast growth factors
FGFR	Fibroblast growth factor receptor
HIF-1α	Hypoxia-inducible factor 1α
HPV	Human papillomavirus
IL	Interleukin
miR-224	Precursor of microRNAs
mTOR	Mammalian target of rapamycin
NF-ƙkB	Nuclear factor-kappa B
Nrf2	Nuclear factor-like 2
p53	Tumor supressor protein
PCT	Procalcitonin
PD-1	Programmed death 1 receptor
PD-L1	Programmed death ligand 1
PSA	Prostate-SpecificAntigen
PTX-3	Pentraxin 3
RANKL	Receptor activator of nuclear factor kappa-B ligand
RELB	Protein coding gene (RELB Proto-Oncogene, NF-ƙKB Subunit)
ROS	Reactive oxygen species
SIR2	Member 2 of sirtuin family
SRC	Proto-oncogene tyrosine kinase
STAT3	Signal transducer and activator of transcription 3
sTRAIL	Soluble TNF-related apoptosis-inducing ligand
suPAR	Soluble urokinase plasminogen activator receptor
TGF-β1	Transforming growth factor β-1
TLR	Toll-like receptors
TNF-α	Tumor necrosis factor α
TrkB	Tropomyosin receptor kinase B
VEGF	Vascular endothelial growth factor
